# Screening for birth-related PTSD: psychometric properties of the Turkish version of the Posttraumatic Diagnostic Scale in postpartum women in Turkey

**DOI:** 10.1080/20008198.2017.1306414

**Published:** 2017-04-03

**Authors:** Pelin Dikmen-Yildiz, Susan Ayers, Louise Phillips

**Affiliations:** ^a^ Centre for Maternal and Child Health Research, School of Health Sciences, City, University London, London, UK

**Keywords:** Post-traumatic stress disorder, postpartum women, PDS, psychometric properties, factor analysis

## Abstract

**Background**: Evidence suggests that 4% of women develop posttraumatic stress disorder (PTSD) after childbirth, with a potentially negative impact on women and families. Detection of postpartum PTSD is essential but few measures have been validated in this population.

**Objective**: This study aimed to examine psychometric properties of the Turkish version of the Posttraumatic Diagnostic Scale (PDS) to screen for birth-related PTSD among postpartum women and identify factorial structure of PTSD after birth.

**Method**: PDS was administered to 829 postpartum women recruited from three maternity hospitals in Turkey. Participants with PTSD (*N *= 68) and a randomly selected group of women without PTSD (*N *= 66), underwent a structured clinical interview (SCID).

**Results**: PDS demonstrated high internal consistency (α = .89) and test-retest reliability between 4–6 weeks and 6-months postpartum (*r_s_ *= .51). PDS showed high concurrent validity with other measures of postpartum psychopathology, *r_s_*(829) = .60 for depression and *r_s_*(829) = .61 for anxiety. Satisfactory diagnostic agreement was observed between diagnoses obtained by PDS and SCID, with good sensitivity (92%) and specificity (76%). Exploratory and confirmatory factor analyses revealed that the latent structure of birth-related PTSD was best identified by a three-factor model: re-experiencing and avoidance (RA), numbing and dysphoric-arousal (NDA) and dysphoric-arousal and anxious-arousal symptoms (DAA).

**Conclusions**: The findings supported use of PDS as an effective screening measure for birth-related PTSD among postpartum women.

## 1. Introduction

Research on posttraumatic stress disorder (PTSD) has shown that the disorder is more widespread than previously estimated and more pronounced in certain populations. Although originally associated with military combat, natural disasters and physical/sexual assault, PTSD following difficult childbirth has now become recognized (Konig et al., 2016; Vossbeck-Elsebusch, Freisfeld, & Ehring, 2014). A woman with a complicated or traumatic birth might present with PTSD symptomatology, by developing re-experiencing, avoidance, numbing and arousal symptoms. Epidemiological studies find prevalence rates of birth-related PTSD of 0–11.6% in community samples (Vossbeck-Elsebusch et al., 2014; Wenzel, Haugen, Jackson, & Brendle, 2005) and 0–43% in high-risk samples including women with preterm birth (Shaw et al., 2014), emergency caesarean section (Ryding, Wijma, & Wijma, 1997) and history of physical/sexual or childhood abuse (Muzik et al., 2013). A recent review and meta-analysis concluded the average prevalence is 4% in postpartum women generally, and 18.9% in high-risk women (Yildiz, Ayers, & Phillips, 2017). Previous research suggests that birth-related PTSD adversely affects women, their infants and the parental relationship, with some evidence of a negative impact on the couples’ relationship (Parfitt, Pike, & Ayers, 2014), bonding difficulties (Nicholls & Ayers, 2007), fetal growth (Koen et al., 2016) and poorer infant cognitive development (Parfitt et al., 2014).

Given the increasing awareness of the occurrence of birth-related PTSD and its negative effects, it is essential to have reliable and culturally valid screening instruments to identify women with PTSD who need treatment. Several PTSD measures have been created and used within different trauma populations in psychological trauma research (Olff, 2015). For birth-related PTSD, the most frequently used self-report measures are the Posttraumatic Diagnostic Scale (PDS; Foa, Cashman, Jaycox, & Perry, 1997), Impact of Events Scale (IES; Horowitz, Wilner, & Alvarez, 1979) and Perinatal PTSD Questionnaire (PPQ; DeMier, Hynan, Harris, & Manniello, 1996). PDS is superior to other measures in providing a complete assessment of PTSD in accordance with the DSM-IV-TR criteria, by assessing all A–F criteria. Due to its diagnostic ability in establishing cases of PTSD along with adaptability for use with different types of traumas, PDS has been used in perinatal research to examine the prevalence or risk factors for PTSD following birth (Shlomi Polachek, Harari, Baum, & Strous, 2012; Vossbeck-Elsebusch et al., 2014). However, the psychometric properties of PDS as a screening tool for birth-related PTSD have not been investigated among postpartum populations. Undoubtedly, further research would allow better understanding of how to assess PTSD in obstetric settings reliably and validly, and be used for both clinical and research purposes.

In Turkey, PDS was translated and adapted in Turkish by Isikli (2006) and found to have acceptable reliability and validity for use in different trauma-exposed samples. It has been used in trauma-focused studies in Turkey with both non-clinical samples (Gul, 2014) and high-risk populations, including women with a history of sexual abuse (Golge, Yavuz, Korkut, & Kahveci, 2013) and emergency service personnel (Baysak, 2010). However, a number of limitations still need to be addressed. First, the Turkish version of PDS has been adapted, but not validated (Golge et al., 2013). Second, the original study adapting PDS was conducted on a relatively small sample (*N* = 90; Isikli, 2006). Third, the performance of PDS in relation to a standardised diagnostic interview and the long-term stability of PDS have not been assessed. Finally, since psychometrics of the scores with target population are more important than psychometrics of a measure (Wilkinson & Inference, 1999), screening tools need to be validated for the targeted population.

The factor structure of PTSD is also contentious and important to address. The identification of the exact latent structure of birth-related PTSD would aid development of a valid instrument and interventions that capture disorder-specific symptoms. Empirical tests in different samples have resulted in many models of PTSD being proposed. The most empirically supported and competing models are the four-factor models of King, Leskin, King, and Weathers (1998) and of Simms, Watson, and Doebbelling (2002) and the five-factor model (Elhai et al., 2011) (Table 1). However, current literature is inconclusive about the latent structure of PTSD (Yufik & Simms, 2010), which is critical in terms of assessment and treatment of PTSD. More importantly, there is only one study that examined the symptom structure of PSTD following birth, which found two factors of intrusions and avoidance (i.e. birth-related symptoms) and numbing and arousal (i.e. general symptoms) (Ayers, Harris, Sawyer, Parfitt, & Ford, 2009).

**Table 1.** PDS item descriptions, Item mapping for the tested models, EFA factor loadings for the Model F, CFA standardized factor loadings and Squared multiple correlations for the Model F.

	Proposed factor structures for PTSD						EFA factor loadings			Communalities		CFA factor loadings			
PDS items	Model A	Model B	Model C	Model D	Model E	Model F	RA	NDA	DAA	In	Ex	RA	NDA	DAA	*R^2^*
B1: intrusive thoughts	R	R	R	R	RA	RA	.**67**	−.05	.13	.58	.61	.72			.52
B2: recurrent dreams	R	R	R	R	RA	RA	.**56**	−.03	.10	.40	.39	.75			.55
B3: flashbacks	R	R	R	R	RA	RA	.**70**	.03	−.16	.37	.36	.64			.41
B4: emotional reactivity	R	R	R	R	RA	RA	.**49**	−.08	.23	.44	.42	.67			.45
B5: physiological reactivity	R	R	R	R	RA	RA	.**73**	.03	−.08	.44	.47	.63			.40
C1: avoidance of thoughts	A	A	A	AN	RA	RA	.**63**	−.04	.30	.75	.73	.89			.80
C2: avoidance of reminders	A	A	A	AN	RA	RA	.**75**	.03	.14	.74	.73	.87			.76
C3: amnesia	N	D	N	AN	RA	RA	.**65**	.10	−.14	.31	.33	.46			.21
C4: loss of interest	N	D	N	AN	NA	NDA	.31	.**56**	−.15	.36	.40		.55		.30
C5: detachment	N	D	N	AN	NA	NDA	.03	.**77**	−.13	.44	.54		.47		.23
C6: feeling numb	N	D	N	AN	NA	NDA	−.00	.**59**	.02	.32	.35		.48		.23
C7: hopelessness	N	D	N	AN	NA	NDA	−.10	.**76**	.08	.48	.59		.70		.49
D1: sleeping difficulty	DA	D	HA	HA	NA	NDA	−.09	.**49**	.38	.42	.46		.69		.47
D2: irritability	DA	D	HA	HA	NA	NDA	−.07	.**54**	.27	.39	.44		.64		.41
D3: difficulty concentrating	DA	D	HA	HA	NA	DAA	−.02	.02	.**77**	.49	.58			.61	.37
D4: overly alert	AA	HA	HA	HA	NA	DAA	.24	−.04	.**58**	.57	.56			.76	.57
D5: easily startled	AA	HA	HA	HA	NA	DAA	.27	−.02	.**53**	.57	.54			.88	.78
Factor correlations in EFA															
RA								.27	.70						
NDA									.38						
Factor correlations in CFA															
RA								.57	.77						
NDA									.45						

PDS = Posttraumatic Diagnostic Scale; EFA = Exploratory factor analysis; Model F = EFA-derived model; CFA = Confirmatory factor analysis; PTSD = Posttraumatic stress disorder; Model A = five-factor model (Elhai et al., 2011); Model B = four-factor model (Simms et al., 2002); Model C = four-factor model (King et al., 1998); Model D = DSM-IV-TR model (APA, 2000); Model E = two-factor model (Ayers et al., 2009); RA = Re-experiencing and avoidance; NDA = Numbing and dysphoric-arousal; DAA = Dysphoric-arousal and anxious-arousal; In = Initial; Ex = Extraction; R^2^ = Squared multiple correlations; R = Re-experiencing; A = Avoidance; AN = Avoidance and numbing; N = Numbing; D = Dysphoria; NA = Numbing and arousal; DA = Dysphoric-arousal; HA = Hyper-arousal; AA = Anxious-arousal. The highest loadings are boldfaced.

In light of these considerations, the main aim of the present study was designed to validate PDS in a postpartum population, evaluate the performance of the Turkish version of PDS in a large sample of postpartum women and determine whether it can be used as a screening tool for birth-related PTSD. The secondary aim was to examine the factorial structure of birth-related PTSD and compare the goodness of fit of the different competing factor models of PTSD that have been validated in other populations.

## 2. Methodology

### 2.1. Study setting and design

The data for this study were collected as part of the Pregnancy and Childbirth in Turkey (PACT) project. Between May 2014 and May 2015, a multicentre longitudinal study was conducted at three maternity hospitals in purposively selected cities of Turkey: Istanbul, Ankara and Izmir. These cities have the highest birth rates in Turkey (TUIK, 2014) and are in different geographical regions so should provide high-representativeness. Women were recruited in pregnancy (*N *= 950), followed up 6-weeks (*N *= 858) and 6-months after birth (*N *= 829) and were required to complete PDS at each assessment point.

At 6-months postpartum, women with PTSD (*n *= 68) along with a randomly selected group of women who did not meet diagnostic criteria on PDS (*n *= 66) were interviewed using a structured clinical interview (SCID). The study protocol was granted by the Research Ethics Committee of City University in the UK, and by Kocaeli University in Turkey.

### 2.2. Study participants and procedure

Participants of the present study consisted of women who had completed PDS at 6-months postpartum (*N *= 829). Inclusion criteria were that women were at least 18 years of age and signed informed consent. Women who experienced late fetal loss, stillbirth or neonatal death were excluded. Since maternity hospitals serve both community and high-risk pregnancies, the sample consisted of women with both low- and high-risk conditions. To assess whether a woman had birth-related PTSD on PDS, exact DSM-IV-TR criteria were followed. For diagnosis of birth-related PTSD, a woman must have met A1 and A2 criteria; developed at least one re-experiencing symptom (B), at least three avoidance symptoms (C) and at least two hyper-arousal symptoms (D); and experienced the disturbance for at least one month (E) and reported impairment in significant areas of functioning (F). Based on these criteria, PDS identified a total of 76 (9.2%) women as having birth-related PTSD at 6-months postpartum. Participants with PTSD (*n* = 76) and a random sample of women (*n* = 76) from the remaining group (*n* = 753) were contacted by phone and invited to have an SCID interview. Eighteen women were excluded due to absence of consent form or withdrawal from the study, resulting in 68 participants with PTSD and 66 women without PTSD having a telephone SCID interview for screening of PTSD 6-months postpartum. The SCID interview was conducted within 15 days of PDS being completed. Birth events that were reported as traumatic included preterm birth, emergency caesarean section, obstetric interventions such as episiotomy and vaginal tear, high-risk to baby or mother, preeclampsia and verbally humiliating or aggressive behaviour of health professionals. Women identified as having PTSD based on the SCID were referred for further psychological examination at relevant maternity hospitals.

### 2.3. Study instruments

#### 2.3.1. Posttraumatic Diagnostic Scale (PDS)

PDS (Foa et al., 1997) is a widely used, reliable and validated screening instrument for PTSD, providing both a diagnosis of PTSD by assessing all DSM-IV-TR criteria (A–F) and symptom severity. PDS lists 17 symptoms (Criteria B, C and D), asks about the experience of traumatic event (Criteria A1 and A2), the duration of disturbance (Criterion E) and the impact of symptoms on functioning (Criterion F). Participants are required to rate the items on a 4-point scale ranging from 0 to 3, which gives a minimum score of 0 and a maximum score of 51. Higher scores indicate more severe PTSD symptoms. PDS demonstrates good psychometric properties in mixed trauma survivors where Cronbach alpha was .92 and test-retest reliability of the total PDS score was .83 (Foa et al., 1997).

The Turkish version of PDS has favourable internal consistency in samples of general trauma survivors (*α* = .93) and good concurrent validity, with high correlations with the Brief Symptom Inventory (*r* = .70), Beck Depression Inventory (*r *= .60) and Beck Anxiety Inventory (*r* = .63) (Isikli, 2006). After birth, PDS was adapted to assess birth-related PTSD, by asking women to complete it in relation to their birth experience and birth-related symptoms of PTSD.

### 2.4. Hospital Anxiety and Depression Scale (HADS)

HADS (Zigmond & Snaith, 1983) was used to measure anxiety and depression. It consists of 14 items, where seven items relate to anxiety and seven items relate to depression. Each item on the questionnaire is scored 0–3 with a possible range of 0–21 for either anxiety or depression, where high scores indicate high levels of psychopathology. Cronbach’s alpha for HADS-A ranged from .68 to .93 and for HADS-D from .67 to .90 (Bjelland, Dahl, Haug, & Neckelmann, 2002). The Turkish version of HADS has been found to have satisfactory psychometric properties (Aydemir, 1997).

### 2.5. Edinburg Postpartum Depression Scale (EPDS)

EPDS (Cox, Holden, & Sagovsky, 1987) was used to examine postpartum depression. EPDS consists of 10 items rated on a 4-point scale, ranging from 0 to 3, with a possible maximum score of 30. Higher scores reflect greater risk for depression. Cronbach’s alpha for EPDS was 0.87 in the original study (Cox et al., 1987). EPDS was validated in Turkish samples and validity and reliability were found to be favourable (Aydin, Inandi, Yigit, & Hodoglugil, 2004).

### 2.6. Structured clinical interview for DSM-IV-TR

SCID (First, Spitzer, Gibbon, & Williams, 1996) is a semi-structured clinical interview which ascertains the presence/absence of a psychological disorder in non-clinical populations. The PTSD module of SCID was used to identify birth-related PTSD and determine convergent validity of PDS. SCID is considered a ‘gold standard’ and suggested for use in clinical settings and research (Lee et al., 2004). SCID has been validated in Turkish samples and demonstrated satisfactory psychometric properties with good validity and reliability (Ozkurkcugil, Aydemir, Yildiz, Esen, & Koroğlu, 1999).

## 3. Statistical analyses

Statistical Program for Social Sciences (SPSS) 22.0 and AMOS version 21 were used for analyses. Descriptive statistics were computed to depict socio-demographic characteristics of the sample. Internal consistency of PDS was evaluated by Cronbach’s alpha coefficient. Spearman correlation coefficient and intra-class correlation coefficient (ICC) were used to assess test-rest reliability for severity of PTSD symptoms. Concurrent validity was tested using Spearman correlation coefficient between PDS and HADS, and PDS and EPDS. Diagnostic accuracy, sensitivity, specificity, positive and negative predictive values (PPV and NPV, respectively) and positive and negative likelihood ratios were calculated. The concordance between PDS and SCID diagnoses was analysed with cross-tabulation and Cohen’s kappa coefficient was computed.

Before conducting factor analyses, the total sample (*N *= 829) was randomly divided into two split-half samples. Exploratory factor analysis (EFA) was conducted on the first split-half sample to explore the possible factor structure of birth-related PTSD, using Principal Axis factoring extraction (PFA) with Promax rotation, given that PFA is the most appropriate method for EFA (Matsunaga, 2010) and PTSD clusters are to some extent related. The criteria of eigenvalue of >1, scree plot and parallel analysis (PA) were used to determine the number of factors to be extracted. Confirmatory factor analysis (CFA) was subsequently performed on the remaining split-half to confirm the EFA-derived model and compare it with other empirically proposed factor structures of PTSD. CFA was completed with Maximum Likelihood Model given its robustness to non-normally distributed data (Olsson, Foss, Troye, & Howell, 2000). Indicators of goodness-of-fit were used to evaluate the hypothesised models including Comparative Fit Index (CFI), Tucker-Lewis Index (TLI), the Goodness-of-Fit Index (GFI), Root Mean Square Error of Approximation (RMSEA), Akaike Information Criterion (AIC) and Standardized Root Mean Square Residual (SRMR). The ranges for each index are provided in Table 5 for ease of interpretation. Women who had PTSD (related to any event) in pregnancy were included in the analyses and regarded as having PTSD if they still met criteria for birth-related PTSD at 6-months postpartum.

**Table 2.** Correlations of the Posttraumatic Diagnostic Scale (PDS) with validity measures (*N* = 829).

Scale	*M*	*SD*	1	2	3	4	5	6	7
1. PDS Total	6.57	7.03	__						
2. PDS Re-experience	2.17	2.50	.64	__					
3. PDS Avoidance	2.19	2.93	.80	.33	__				
4. PDS Hyper-arousal	2.19	2.60	.77	.27	.52	__			
5. EPDS Total	7.86	5.07	.60	.31	.56	.46	__		
6. HADS Total	11.93	6.78	.61	.29	.58	.47	.68	__	
7. HADS Anxiety	7.16	3.83	.49	.32	.42	.39	.54	.83	__
8. HADS Depression	4.77	4.26	.52	.19	.54	.38	.59	.84	.43


All correlation coefficients were significant, *p* < .001. EPDS = Edinburgh Depression Scale; HADS = Hospital Anxiety and Depression Scale.

**Table 3.** Agreement of PDS and SCID diagnoses for birth-related PTSD.

	SCID PTSD diagnosis		
PDS diagnosis of PTSD	Absent	Present	Total
Negative	62 (46.3%)	4 (3.0%)	66 (49.3%)
Positive	20 (14.9%)	48 (35.8%)	68 (50.7%)
Total	82 (61.2%)	52 (38.8%)	134 (100.0%)

**Table 4.** Psychometric properties of PDS relative to the SCID diagnosis of PTSD after birth.

Statistical measures	PDS DSM-IV-TR diagnostic criteria	95% CI
Sensitivity (%)	92	80.5–97.5
Specificity (%)	76	64.6–84.1
False-positive rate (%)	24	14.8–33.2
False-negative rate (%)	8	.6–15.4
Positive predictive value (%)	70	58.1–80.7
Negative predictive value (%)	93	84.4–98.0
Overall accuracy (%)	82	69.5–94.5
Kappa (%)	64	51.7–76.9
Positive likelihood ratio	3.78	2.56–5.58
Negative likelihood ratio	.10	.03–.26

**Table 5.** Fit indices for the six proposed models.

Factor model	χ^2 (a)^	*Df*	CFI^(b)^	TLI^(c)^	GFI^(d)^	RMSEA^(e)^	SRMR^(f)^	AIC^(g)^
Five-factor model (Elhai et al., 2011)	330.83	109*	.93	.91	.92	.070	.071	418.83
Four-factor model (Simms et al., 2002)	352.95	113*	.92	.91	.91	.072	.070	432.95
Four-factor model (King et al., 1998)	493.38	113*	.88	.85	.86	.090	.081	573.38
DSM-IV-TR model (American Psychiatric Association, 2000)	607.95	116*	.84	.81	.82	.101	.085	681.95
Two-factor model (Ayers et al., 2009)	576.73	118*	.85	.82	.83	.097	.074	646.73
EFA-derived model	294.75	116*	.94	.93	.92	.061	.053	368.75

^(a)^ The *χ* ^2^ values for all models were significant suggesting that a substantial proportion of the variance is unexplained by the model (Kline, 2005); ^(b)^ CFI = Comparative Fit Index; ^(c)^ TLI = Tucker-Lewis Index; ^(d)^ GFI = Goodness-of-Fit Index; ^(e)^ RMSEA = Root Mean Square Error of Approximation; ^(f)^ SRMR = Standardized Root Mean Square Residual; ^(g)^ AIC = Akaike Information Criterion. CFI, TLI and GFI values > .9 indicates a good fit; SRMR and RMSEA < .08 indicates an adequate fit (Hu & Bentler, 1999). The model with the lowest AIC was considered to be the best-fit model among others. * *p* < .001.

## 4. Results

### 4.1. Sample characteristics

A total of 829 mothers completed PDS at 6-months postpartum. The mean age of participants was 27 years (*SD* = 5.31; range, 18–44 years); 360 (43.4%) were pregnant for the first time; 681 (82.1%) planned the current pregnancy; 825 (99.5%) were married; and 150 (18.1%) were employed. The sample was broadly representative of Turkish women in terms of level of education with 133 (16%) women with university degrees or above.

### 4.2. Proportion of women having PTSD or symptoms at 6-months postpartum

Seventy-six women (9.2%) met full diagnostic criteria for birth-related PTSD 6-months postpartum based on PDS. More than one-third of women met A1 criteria (35.2%) and two-thirds of women met A2 criteria (69.7%). In accordance with DSM-IV-TR criteria, 75% of women reported at least one re-experiencing symptom, 22% reported at least three avoidance symptoms and 47% reported hyper-arousal symptoms. Of women who had PTSD at 6-months postpartum (*n* = 76), 31 women met criteria for PTSD at 4–6 weeks postpartum as well. Although 49 women had PTSD in pregnancy, 12 reported PTSD in relation to birth and their symptoms persisted to 6-months postpartum.

### 4.3. PDS scores by diagnostic groups at 6-months postpartum

**Figure 1.**
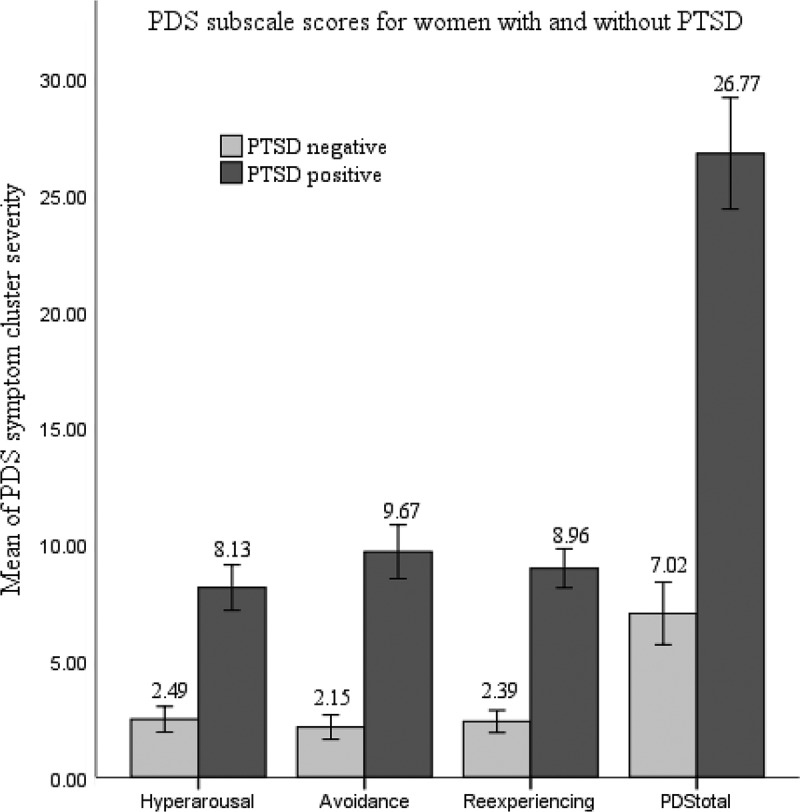
Group differences in PDS and its subscales between women with PTSD (*n* = 52) and without (*n* = 82) diagnosed by SCID. Bars represent means of total symptom severity for PDS and each subscale.

Means and standard deviations for PDS symptom severity and the three subscales were calculated for the whole sample (Table 2). Figure 1 also presents the means of PDS symptom severity for women who had positive and negative SCID-diagnosis for PTSD. It is notable that the means of women diagnosed with PTSD according to SCID were significantly higher for PDS total and subscale scores than those of women who did not meet PTSD criteria on the SCID. The effect sizes (Cohen’s *d*) for each of these mean differences were reasonably large: 2.75 for total symptom severity, 2.59 for Re-experiencing subscale, 2.37 for Avoidance subscale and 1.93 for Hyper-arousal subscale.

### 4.4. Internal consistency

Cronbach’s alpha for PDS scores at 6-months postpartum was .89. The average item-total correlation was .54, ranging from *r* = .33 (Item 11: Feeling emotionally numb) to *r* = .74 (Item 6: Trying not to think about, talk about or have feelings about the birth). Alpha coefficients for the re-experiencing, avoidance and arousal subscales were .80, .75 and .73, respectively.

The subscales and the total PDS scores were highly correlated with each other in the total sample (see Table 2).

### 4.5. Test-retest reliability of PDS symptom severity scores

The stability of PDS symptom severity scores was assessed over a 5-month period. Moderate to large correlations were found between scores obtained at 4–6 weeks and 6-months postpartum, for PTSD symptom severity total score (*r_s_ *= .51, *p* < .001), and for re-experiencing (*r_s_ *= .55, *p* < .001) and avoidance (*r_s_* = .43, *p* < .001). A smaller correlation was found for the arousal subscale (*r_s_* = .25, *p* < .001). The ICC value was also .74 (*p* < .001), indicating a moderate degree of reliability.

### 4.6. Test-retest reliability of PTSD diagnoses

The test-retest reliability of PTSD diagnoses on PDS was examined by kappa as a chance-corrected measure of agreement. There was moderate agreement between PDS diagnoses at 4–6 weeks and 6-months postpartum with a kappa value of .43 [95% CI 0.32, 0.52], *p* < .001. The percent in agreement between diagnoses at the two time points was 89%.

### 4.7. Concurrent validity

Concurrent validity was assessed to determine the extent to which PDS scores correlated with measures of anxiety (HADS) and depression (EPDS). PDS was found to have high levels of concurrent validity with well-validated measures of anxiety and depression. Table 2 illustrates the correlation of PDS and PDS cluster scores with other measures of postpartum psychopathology.

### 4.8. Convergent validity

**Figure 2.**
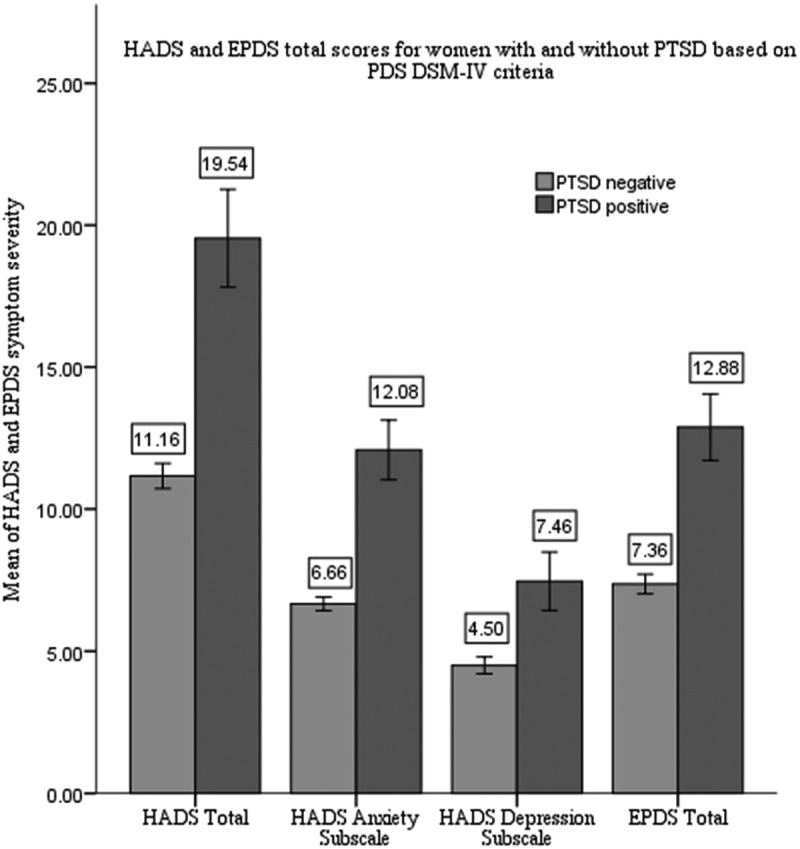
Group differences between participants with and without PTSD according to PDS at 6-months postpartum. Group differences in EPDS and HADS along with its subscales between women with PTSD (*n* = 94) and without (*n* = 735) diagnosed by PDS. Bars represent means of total symptom severity for EPDS and HADS.

Women with PTSD based on PDS had higher depression scores on both EPDS and HADS than participants without PTSD (Figure 2), which supported the convergent validity of PDS.

### 4.9. Comparison of PDS and SCID diagnoses via cross-tabulation

Comparison of diagnoses based on PDS and SCID showed that 52/134 (38.8%) fulfilled the diagnostic criteria on SCID and 68 (50.7%) met criteria on PDS (see Table 3).

Forty-eight (92%) of the 52 women meeting criteria on SCID also met criteria on PDS. Conversely, 62 of the 82 women who failed to meet criteria on SCID were also identified as negative on PDS. A kappa value of .64 between PDS and SCID was obtained, with 82% agreement between the two measures. These analyses provided further evidence for convergent validity with acceptable values of sensitivity, specificity, accuracy, PPV, NPV and likelihood ratio values (Table 4), establishing a preliminary basis for the usefulness of the Turkish version of PDS in clinical settings.

### 4.10. Exploratory factor structure of PTSD

Exploratory factor analysis was conducted on the first split-half of the sample. The Kaiser-Meyer-Olkin (KMO) test showed the data were suitable for factor analysis (.91), and the Barlett’s test was significant (*p* < .001) indicating that the factor analysis is suitable for the given study. A rotated factor solution for PDS revealed three-factor structure with eigenvalues >1.0, accounting for 58.8% of variance. The results of parallel factor analysis also supported the three-factor solution. All factor loadings were greater than .45, ranging from .49 to .77 (Table 1). The first factor accounted for 38.3% of variance and included re-experiencing and avoidance symptoms (RA). The second factor accounted for 14.3% of variance and consisted of numbing and dysphoric-arousal symptoms (NDA). The third factor accounted for additional 6.4% of variance and comprised of a dysphoric-arousal symptom and anxious-arousal symptoms (DAA). The correlations among the three factors were relatively moderate to large (Table 1).

### 4.11. Comparison of factor models via confirmatory factor analyses

CFA was performed on the second split-half sample to cross-validate the EFA-derived model and to test the goodness of fit of the model identified here and other models identified in previous literature (Table 1). Given the fit statistics (Table 5), the EFA-derived three-factor model provided the best fit to the data with the highest CFI, TLI and GFI values and lowest RMSEA. Notably, the five-factor model of Elhai et al. (2011) also achieved acceptable fits. The four-factor model of Simms et al. (2002) performed slightly better than the model of King et al. (1998) and approached a good fit to the data. Although the model of Ayers et al. (2009) derived a similar sample of postpartum women, the fit indices for this model were not within the recommended range according to CFA. The DSM-IV-TR model was the most unfavourable model. Standardized factor loadings were greater than .45, ranging from .46 to .89 (Table 1).

## 5. Discussion

The present study examined the reliability and validity of the Turkish version of PDS in a cohort of women at 6-months postpartum. The aims were to establish the psychometric properties of PDS in postpartum women and to evaluate the structure of birth-related PTSD. The results provided preliminary support for the use of PDS as a useful screening tool for birth-related PTSD among postpartum women and for a three-factor model of birth-related PTSD.

Overall, the reliability of the Turkish version of PDS was favourable in postpartum women. Internal consistency was high and similar to that of the original version (Foa et al., 1997). Average total item correlations also corresponds with those found in the earlier version of PDS, with correlations of .54 in this study versus their average correlations of .45, respectively.

Test-retest analysis suggested the stability of PDS symptom severity and diagnosis over time was lower for postpartum women compared to results from other studies (Dragan, Lis-Turlejska, Popiel, Szumiał, & Dragan, 2012; Powers, Gillihan, Rosenfield, Jerud, & Foa, 2012). However, in the current study test-retest reliability of PDS symptoms was assessed over a 5-month span which is substantially longer than the 1-month period used by those studies. A study of test-retest reliability of the PTSD Symptom Scale (a precursor to PDS) over a 6-month period found similar rates to that found in this study (Stieglitz, Frommberger, Foa, & Berger, 2001).

PDS had good concurrent validity with measures of anxiety (HADS) and depression (EPDS). The correlation between PDS and depression corresponds well with that found in other studies of PDS with postpartum women (Parfitt & Ayers, 2009; Schwab, Marth, & Bergant, 2012). The correlation between PDS and anxiety was of a similar magnitude to that reported in studies of non-postpartum populations (Chung, Jones, Harding, & Campbell, 2015; Sumpter & McMillan, 2005). Foa et al. (1997) found that depression is more correlated with avoidance and hyper-arousal subscales of PDS than the intrusion subscale, which was also reflected in this study.

For convergent validity, the findings are promising and consistent with the existing literature. A kappa value .64 was very close to the kappa coefficient (*κ* = .65) found by Foa et al. (1997) and higher than PDS-SCID concordance reported by Powers et al. (2012) and Sheeran and Zimmerman (2002). The Turkish version of PDS showed satisfactory levels of sensitivity and specificity, which exceeded those obtained by Foa et al. (1997), Powers et al. (2012) and Sheeran and Zimmerman (2002). The high sensitivity of PDS indicated that most women with PTSD due to birth according to SCID were correctly identified by PDS. This greater sensitivity is essential for a screening instrument because it does not allow positive cases to be missed. However, in many clinical contexts, specificity may be prioritised over sensitivity, particularly where resources are limited and cost is a concern. Although the specificity (76%) observed in this study was lower than the sensitivity, it is deemed optimal in most cases. Notably, all women with PTSD diagnosed by SCID had mean PDS scores higher than the minimum recommended cut-off score of 15 and equivalent to the empirically derived cut-off score of 27 (Sheeran & Zimmerman, 2002). Likewise, PTSD positive cases identified by PDS showed greater anxiety and depression compared to women without PTSD. Thus, the results suggest that PDS is a valid screening measure for birth-related PTSD.

The present study also provided insight into factorial structure of birth-related PTSD. Both EFA and CFA supported a three-factor solution which was different to the most supported models of PTSD in the broader literature (Elhai et al., 2011; King et al., 1998; Simms et al., 2002). This may be due to a number of factors. Birth is qualitatively different from other types of traumatic events in terms of predictability, being entered into voluntarily and culminating in the birth of a baby (Ayers, Joseph, McKenzie-McHarg, Slade, & Wijma, 2008), which might contribute to different structure of PTSD. Alternatively, this might reflect cultural differences in the expression of PTSD. The previously proposed model of birth-related PTSD based on a UK sample was also not consistent with the three-factor solution found in the present study, highlighting how difficult it is to determine the exact structure of PTSD, even if the type of trauma is identical.

The first factor of the three-factor solution consisted of re-experiencing and avoidance symptoms, which is consistent with some studies (Buckley, Blanchard, & Hickling, 1998; Taylor, Kuch, Koch, Crockett, & Passey, 1998) and a theoretical framework (Foa, Riggs, & Gershuny, 1995). This theoretical model assumes that re-experiencing symptoms culminate in avoidance symptoms, which might explain the coherence between the two in the current study. The second factor was numbing and dysphoric-arousal symptoms, which may correspond to non-specific symptoms of birth-related PTSD, reflecting general distress. The third factor included concentration difficulties, being overly alert and easily startled. This may represent a unique latent construct separate from the two symptom clusters in women experiencing traumatic births. However, the model was limited to the sample of women involved in this study. Further research is required to examine whether support for this model is found in women after birth so that interventions can be targeted at those symptom clusters that are associated with poorer outcomes.

All communalities were relatively moderate to high except the item of trauma-related amnesia. The amnesia symptom attributed to PTSD might be primarily related to dissociation and may only occur for the dissociative subtype of PTSD (Lanius, Brand, Vermetten, Frewen, & Spiegel, 2012), which may not be highly applicable to birth-related PTSD. In the present study, 68% of women did not reveal any symptoms of amnesia. Similarly, in a study by Shlomi Polachek et al. (2012), none of the women reported any amnesia during birth.

High inter-correlations between items C1 (avoidance of thoughts) and C2 (avoidance of reminders), and D4 (overly alert) and D5 (easily startled), were observed, which is indicative of either a common theme that they do not share with other items or the simultaneous occurrence of these PTSD symptoms. This result supports DSM-5 formulation of PTSD where Avoidance now represents its own cluster and arousal symptoms remain relevant. Since there is currently no self-diagnostic measure of birth-related PTSD according to DSM-5 criteria available, PDS was used in this study. Further research needs to determine how to best measure birth-related PTSD and subsequently the latent structure given the DSM-5 criteria.

The present study has limitations that should be taken into consideration when interpreting the results. All participants were postnatal women; thus, the results may not be generalized to other samples. Since women were recruited from cities located in the West and Central regions of Turkey, it is unclear how generalizable the sample is to postpartum women in different parts of Turkey who might have different postpartum traditions and practices. Discriminant validity was not assessed; although it could have contributed to the strong psychometric properties of PDS. DSM-IV-TR criteria was used in PTSD case identification, yielding no empirically derived PDS cut-off score for different purposes and groups. SCID interviews were conducted by a researcher who was not blind to each woman’s PDS scores, so interviewer bias may have affected assessment of PTSD diagnoses. Lastly, DSM-IV nomenclature underwent revision over the duration of study which limits the applicability of the results to DSM-5.

Despite these limitations, this unique study represents the first attempt in demonstrating the reliability and validity estimates of PDS among the postpartum population. It is only the second investigation examining the factor structure of birth-related PTSD. By using a large sample size, a variety of measures and complementary statistical analyses, this study validates the use of PDS to screen for birth-related PTSD in a sample of postpartum women in Turkey for the first time.

The findings of this study have important clinical implications, especially given that PTSD after birth is associated with adverse consequences for both women and their baby. It is therefore crucial to identify women at risk of, or suffering from, PTSD after birth and screening tools are an important first step to identify women who may need further assessment and/or treatment. Future research is warranted to examine whether DSM-5 criteria are also coherent with postpartum samples and to determine which cut-off scores are most predictive of distress and need for treatment.

## 6. Conclusion

PDS was found to be an effective screening tool for birth-related PTSD among postpartum women in Turkey. Psychometric properties of the Turkish version of PDS were as good as those reported for other populations and languages. This suggests that PDS can be used as a screening instrument to identify PTSD following birth in a valid and reliable way. It is expected that this study will stimulate further research in establishing the reliability and validity of PDS among postpartum women in different cultures for eventual use in all perinatal populations.

## Highlights

- Few measures of PTSD have been validated among postpartum women.
- The Turkish version of PDS demonstrated good psychometric properties.
- PDS was able to detect PTSD after birth.
- A differing three-factor model of PTSD was identified.
- PDS can be used as a screening measure for birth-related PTSD.
